# LncSSBP1 Functions as a Negative Regulator of IL-6 Through Interaction With hnRNPK in Bronchial Epithelial Cells Infected With *Talaromyces marneffei*

**DOI:** 10.3389/fimmu.2019.02977

**Published:** 2020-01-10

**Authors:** Yinghua Li, Huan Chen, Shuyi Li, Yu Li, Guangnan Liu, Jing Bai, Honglin Luo, Xiuwan Lan, Zhiyi He

**Affiliations:** ^1^Department of Pulmonary and Critical Care Medicine, Second Affiliated Hospital of Guangxi Medical University, Nanning, China; ^2^Department of Pulmonary and Critical Care Medicine, Sixth Affiliated Hospital of Guangxi Medical University, Yulin, China; ^3^Guangxi Colleges and Universities Key Laboratory of Preclinical Medicine Research, Guangxi Medical University, Nanning, China; ^4^Department of Pulmonary and Critical Care Medicine, First Affiliated Hospital of Guangxi Medical University, Nanning, China; ^5^School of Basic Medicine, Guangxi Medical University, Nanning, China

**Keywords:** microbial immunology, fungus, *Talaromyces marneffei*, lncRNA, IL-6, hnRNPK, bronchial epithelial cell

## Abstract

*Talaromyces marneffei* (TM) is an important opportunistic pathogenic fungus capable of causing disseminated lethal infection. In our previous study, we identified host lncRNAs and mRNAs that are dysregulated in TM-infected bronchial epithelial cells. In this report, we verified that IL-6, a key factor in acute inflammatory response, is down-regulated in TM pathogenesis. To elucidate the mechanism of IL-6 regulation, we analyzed the coding/non-coding network, and identified lncSSBP1, a novel lncRNA that is up-regulated by TM. Our results demonstrate that overexpression of lncSSBP1 decreases IL-6 mRNA expression, whereas knockdown of lncSSBP1 enhances IL-6 mRNA expression. Though lncSSBP1 is primarily localized to the nucleus, bioinformatics analysis suggests that it is unlikely to function as competing endogenous RNA or to interact with IL-6 transcription factors. Instead, RNA pull down and RNA immunoprecipitation assays showed that lncSSBP1 binds specifically to heterogenous nuclear ribonucleoprotein K (hnRNPK), which is involved in IL-6 mRNA processing. Our findings suggest that lncSSBP1 may affect IL-6 mRNA expression during TM infection through interaction with hnRNPk in bronchial epithelial cells. Our results suggest a novel pathway by which TM may suppress the immune response to its advantage.

## Introduction

*Talaromyces marneffei* (TM), formerly known as *Penicillium marneffei*, is a prominent opportunistic pathogen capable of causing disseminated lethal infection. This thermal dimorphic pathogenic fungus infects immunocompromised hosts including those with acquired immunodeficiency syndrome (AIDS) (1). Disseminated TM is mainly endemic to southern China, Hong Kong, Taiwan and in southeastern Asia (2). In mainland China, the majority of TM infection cases are reported in southern China, and more than 80% of them are from Guangdong and Guangxi Provinces (3). With increased immunodeficiency and immunocompromise, the incidence of TM infection has been steadily increasing, leading to ~50,000 new cases of TM infections diagnosed each year (4). In endemic areas, TM together with tuberculosis and cryptococcosis, are the top three opportunistic infections in AIDS patients (5). Current predictions suggest that 16.1% of AIDS patients in Guangxi Province suffer from TM infection, and the mortality rate of TM infection is significantly higher than that of any other AIDS complication (6). If appropriate systemic antifungal therapy is not administered in a timely fashion, the mortality rate can reach as high as 33% (7). Furthermore, the recurrence rate of disseminated TM is up to 50% (8).

In the course of a typical TM infection, TM conidia are inhaled into the lower respiratory tract, adhere and penetrate to bronchial epithelial cells, are phagocytized by alveolar macrophages, and lurk in the reticuloendothelial system (9). The conidia can disseminate around the body through the reticuloendothelial system (10). Timely initiation and activation of the innate immune response are essential for the host to clear the pathogen. As an important part of the innate immune system, bronchial epithelial cells play a key role in preventing pathogen invasion and inducing subsequent immune responses and immune evasion (11). Therefore, increased understanding of the response of epithelial cells to TM provides a key strategy for developing novel therapies to combat TM pathology and spread.

IL-6, a critical pleiotropic molecule in inflammation, imparts context-dependent pro-inflammatory and anti-inflammatory properties (12). Binding of IL-6 to the IL-6 receptor mediates intracellular signal transduction pathways, which often serve to boost innate immune responses during pathogenic infections (13). Recent studies have shown that IL-6 activates the classic signaling pathway via membrane bound IL-6 receptors and the trans-signaling pathway via soluble IL-6 receptors (12–14). Classic signaling is mainly considered to be protective and regenerative (anti-inflammatory), while trans-signaling is considered pro-inflammatory. Therefore, IL-6 is an important regulator of the balance between pro-inflammatory and anti-inflammatory responses that may be modulated by pathogens to temper the host's response.

In our previous study, to reveal the relationships between TM and lncRNAs, we used microarray to profile dysregulated lncRNAs and mRNAs in a human bronchial epithelial cell model that was infected with TM conidia for 4 h, including IL-6, which was down-regulated by TM (15). In this report, we have extended these findings by demonstrating that lncSSBP1 negatively regulates IL-6 expression in TM-infected bronchial epithelial cells. Furthermore, we demonstrate that heterogenous nuclear ribonucleoprotein K (hnRNPK) serves as a binding partner of both lncSSBP1 and IL-6 mRNA. Therefore, our results elucidate a pathway by which lncSSBP1 may regulate innate immunity in TM-infected cells.

## Materials and Methods

### Compliance With Ethical Standards

The TM strain was isolated from the sputum an HIV-negative patient suffering from disseminated TM infection at the first affiliated hospital of Guangxi Medical University, as described in our previous study (15, 16). The isolation was a part of standard care of the patient. Subsequent isolation of the microorganism was undertaken according to standard laboratory processes. Our study was approved by the First Affiliated Hospital of Guangxi Medical University Ethical Review Committee.

### TM Conidia Preparation

The strain was cultured on potato dextrose agar medium (Luqiao Technology, Beijing, China) at 25°C for 7–10 days. Colonies were washed with sterile phosphate buffed saline, and then conidia were collected by centrifugation.

### Cell Culture and Infection

The human bronchial epithelial cell line BEAS-2B was preserved in our laboratory, Guangxi Medical University, Guangxi, China. Cells were cultivated in RPMI1640 medium mixed with 10% fetal bovine serum (Invitrogen, Carlsbad, USA), and were placed in cell incubator at 37°C with 5% CO_2_. To initiate infection, BEAS-2B cells were stimulated with TM conidia for 4 h.

### Transmission Electron Microscopy

Cells were fixed with 1% glutaraldehyde and 4% paraformaldehyde in PBS at 4°C overnight followed by post-fixation in 1% buffered osmium tetroxide. Cells were dehydrated through a graded series of ethanol (30, 50, 70, 80, 90, and 100%) for 10 min each. The cells were then embedded in epoxy resin and polymerized. After polymerization, the embedded cells were cut into ultrathin sections using an ultramicrotome (Leica, Wetzlar, Germany), and stained with 1% uranyl acetate followed by lead citrate. All samples were viewed on a transmission electron microscope (Hitachi H-7650, Tokyo, Japan).

### RNA Extraction and qRT-PCR

Total RNA was extracted from cells from both the control and treatment groups with TRIzol reagent (Invitrogen, Carlsbad, USA), following the manufacturer's instructions. The RNA quality and quantity were measured with a Nucleic Acid Protein Detector (Thermo Fisher Scientific, Waltham, USA). Total RNA was used to transcribe into cDNA with the First Strand cDNA Synthesis Kit (TaKaRa, Dalian, China). PCR reactions were run using the following profile: 1 cycle at 95°C for 30 s; 40 cycles of 95°C for 3 s and at 60°C for 30 s. The primers used for qRT-PCR are listed in Table S1. GAPDH expression served as the internal control, and the relative expression levels of lncRNA and mRNA were determined using the 2^−ΔΔCt^ analysis method (17).

### Measurement of IL-6

Levels of IL-6 in cell supernatants were determined using enzyme-linked immunosorbent assay (ELISA) kits (Cat #CSB_E04638h; CUSABIO, Wuhan, China) according to the manufacturer's instructions.

### Construction of a Coding/Non-coding Gene Co-expression Network

A coding/non-coding co-expression network (CNC network) was constructed based on our existing microarray data (15). The Pearson correlation coefficient (PCC) between IL-6 and differentially expressed lncRNAs was calculated with |PCC| ≥ 0.91 and *P* < 0.05 considered meaningful, and then the CNC network was constructed using Cytoscape (v2.8.1). Co-expression relationships with significant *P*-values within a given upper percentile were entered as edges, while IL-6 and lncRNAs were entered as nodes in the CNC network. The co-expression relationship of each gene pair was estimated by the PCC.

### Fluorescence *in situ* Hybridization (FISH) of lncSSBP1 in BEAS-2B Cells

Locked Nucleic Acid (LNA)-based probes were directed against the full length lncRNA sequence for lncSSBP1. The lncSSBP1 probe (sequence: 5′-TAAGAGTTGCTGCCAAGTATTTTCAAAATC-3′) was purchased from BioSense (Guangzhou, China). The FISH procedure was performed according to the BioSense instructions. Briefly, cell slides were fixed in 4% formaldehyde for 20 min and then washed twice for 5 min each with 0.1% diethy pyrocarbonate water. After digesting with proteinase K for 10 min, the slides were fixed in 1% formaldehyde for 10 min. The slides were sequentially subjected to dehydration using 70, 85, and 100% alcohol for 5 min each. Then, the slides were hybridized with the probes overnight (12–16 h) at 45°C in a humidified chamber. At the end of the hybridization period, the slides were sequentially treated with a warmed hybridization solution. Finally, the slides were counterstained with DAPI (Sigma, Shanghai, China) and scanned with Zeiss LSM 700 Meta confocal microscope (Oberkochen, Germany).

### Construction of ceRNA Network

The interactions between lncSSBP1 and miRNA were predicted using DIANA-LncBase v2.0. Then the predicted targets were intersected with differentially expressed genes in the microarray data mentioned above. The lncSSBP1, miRNAs, and mRNAs were selected to construct the lncRNA-miRNA-mRNA regulatory network. The interactions and visualization were conducted by the Cytoscape software v3.4.0.

### Overexpression and Knock Down of lncSSBP1

A lentiviral vector containing human lncSSBP1 was purchased from GenePharma (Suzhou, China) and used to overexpression lncSSBP1 (referred to as Lv-lncSSBP1). The negative control lentivirus (Lv-NC) was also purchased from GenePharma. An shRNA lentivirus vector containing the target sequence of lncSSBP1 (5′-GGCGACAAGCAACAACAATCA-3′) was also used to knock down lncSSBP1 expression. The sequence was cloned into pGLVH1 (GenePharma, SuZhou, China) to generate Sh-lncSSBP1. A negative control lentivirus containing a non-targeting shRNA sequence (5′-TTCTCCGAACGTGTCACGT-3′; referred to as Sh-NC) was used as a control. All cloned sequences were verified by automated sequencing (GenePharma, SuZhou, China).

All lentiviral vectors, including Lv-lncSSBP1, Lv-NC, sh-lncSSBP1 and sh-NC, were transfected into 293FT cells for packaging. The virus particles were harvested 72 h after transfection of 293FT cells. For stable transfection, BEAS-2B cells were grown in six-well plates to 50% confluence, and 1 mL of viral supernatant was added with 1 μL polybrene. The interference efficiency of the template was verified by RT-PCR analysis.

### RNA Pull-Down and Mass Spectrometry Analyses

LncSSBP1 template DNA was transcribed *in vitro* with T7 RNA polymerase (Roche, Basel, Switzerland) and purified with an RNeasy Mini Kit (Qiagen, Dusseldorf, Germany) according to the manufacturer's instructions. The purified 3′ end region of RNA was biotinylated using Pierce™ RNA 3′ End Desthiobiotinylation Kit (Thermo Fisher Scientific, Waltham, USA). Biotinylated RNA was incubated with streptavidin-agarose beads for 2 h at room temperature. Then the beads were washed briefly three times, and the retrieved protein was detected by spectrometry (Sagene Biotechnology, Guangzhou, China).

### RNA Immunoprecipitation Assays

RNA immunoprecipitation was performed according to the instructions of the Magna RIP™RNA-Binding Protein Immunoprecipitation Kit (Millipore, Billerica, MA, USA). Cells were collected using a cell scraper and lysed using RIP lysis buffer containing proteinase inhibitors and ribonuclease inhibitors. Subsequently, whole cell lysates were mixed with magnetic beads conjugated with human anti-hnRNPK antibody (Abcam, Shanghai, China), or negative control IgG (Abcam, Shanghai, China) at 4°C overnight. After immunoprecipitation, the protein A/G-beads were washed with washing buffer. Finally, the immunoprecipitation products were collected. Immunoprecipitated RNA was purified and then subjected to qRT-PCR analysis to detect the relative levels of lncSSBP1 and IL-6. 7SK expression served as a positive internal control.

### Statistical Analysis

Results are shown as the mean ± standard deviation (SD) of three independent experiments for each group. Statistical comparisons were conducted using SPSS20.0, and significance was assessed by the two-tailed Student's *t*-test. Results with *P* < 0.05 were considered statistically significant.

## Results

### TM Infection Induces Morphological Changes in BEAS-2B Bronchial Epithelial Cells

**Figure 1:**
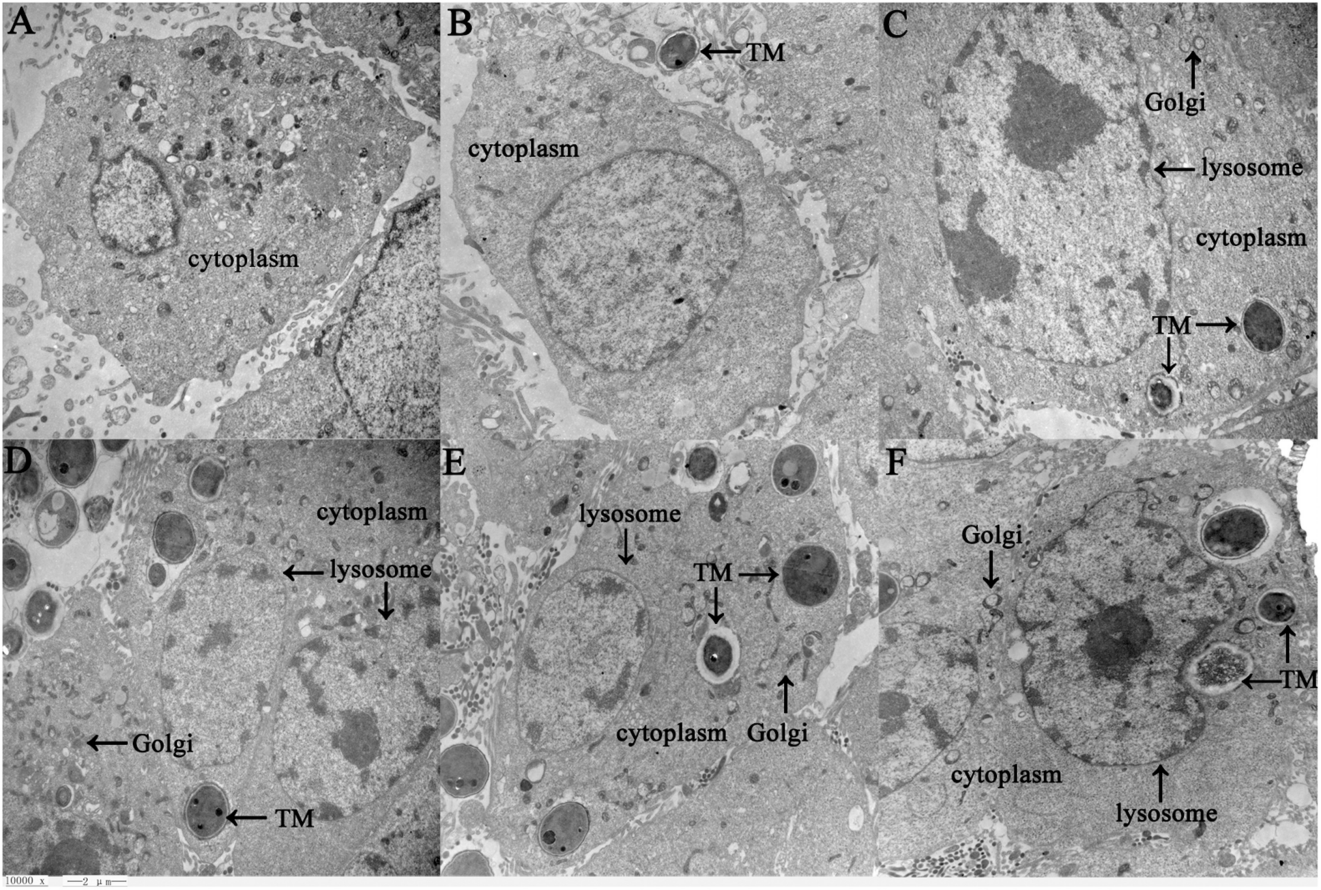
Ultrastructure of BEAS-2B cells in response to TM infection as detected by transmission electron microscopy (10,000 x, Bar = 2 μm). BEAS-2B cells were incubated with TM spores for 1 h **(B)**, 2 h **(C)**, 3 h **(D)**, 4 h **(E)**, or 5 h **(F)**, and then changes between control cells **(A)** and treatment cells **(B–F)** were observed under transmission electron microscopy. Swollen organelles are marked with arrows.

Human bronchial epithelial BEAS-2B cells were incubated with TM conidia at a ratio of 1:20 for 1–5 h, and then observed under a transmission electron microscope. After co-culture for 2 h, TM spores were phagocytized by BEAS-2B cells. With prolonged culture time, the number of phagocytic spores increased, and the phagocytic vacuoles became aggregated. Over time, the amount of intracellular cytoplasm and organelles increased significantly, and the Golgi, lysosomes and other organelles appeared swollen. Pseudopods extended out of the lysosomes, and the spores appeared damaged to a certain degree (Figure 1). These findings verify that TM induces observable pathological changes in a human bronchial epithelial cell culture model.

### Expression of lncRNA NR_046269 and IL-6 Are Negatively Correlated in the Response to TM Infection

**Figure 2:**
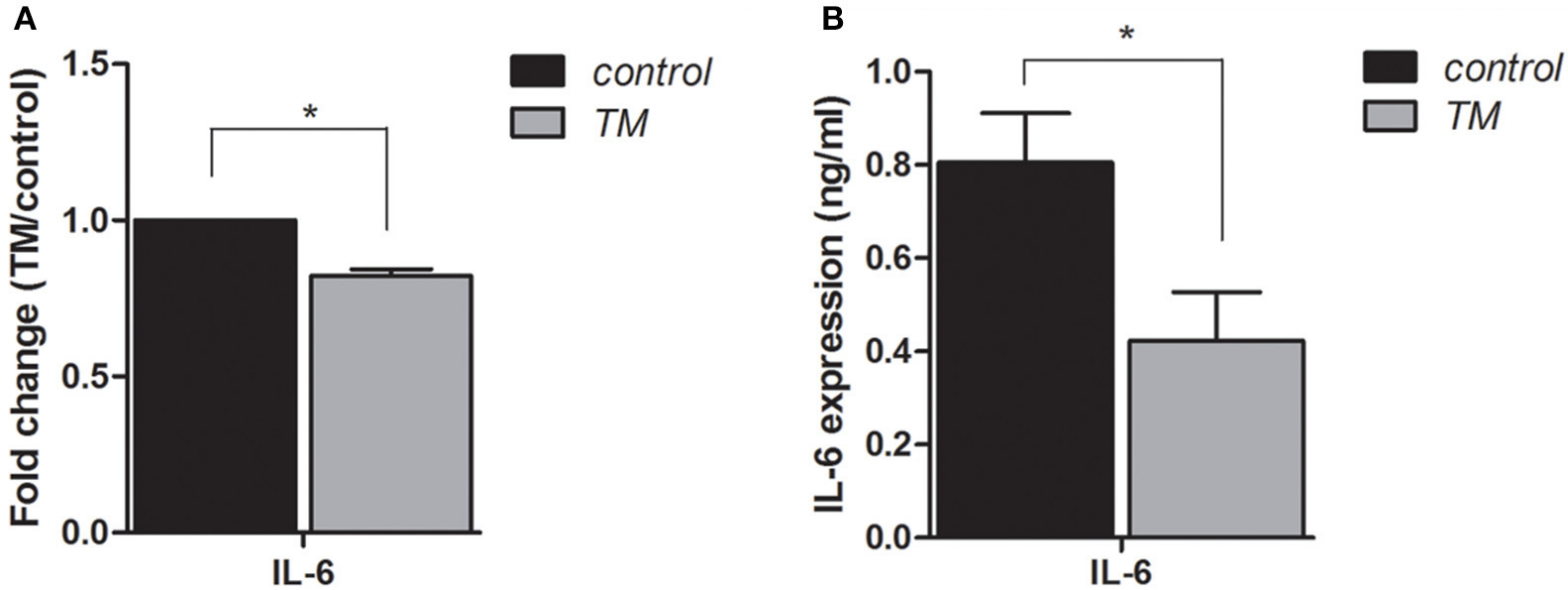
Expression levels of IL-6 in BEAS-2B cells are down-regulated by infection with TM for 4 h. qRT-PCR **(A)** and ELISA **(B)** were performed to determine the expression levels of IL-6, *P < 0.05.

To identify regulatory pathways that may contribute to the pathogenic response to TM infection, we re-examined the lncRNA and mRNA expression profiles from our previous study (15), in which BEAS-2B cells were infected by TM spores for 4 h. We noted that among 519 lncRNAs and 329 mRNAs that were significantly differentially expressed (Fold change ≥ 1.5 or ≤ –1.5, *P* < 0.05). Of these differentially expressed genes, we found that IL-6 expression was down-regulated. Given the key role of IL-6 in mediating inflammatory pathways and processes (12–14), we further examined its expression pattern by qRT-PCR and ELISA. The results verified that the expression of IL-6 is down-regulated in TM-infected cells, which is consistent with microarray data (Figure 2).

**Figure 3:**
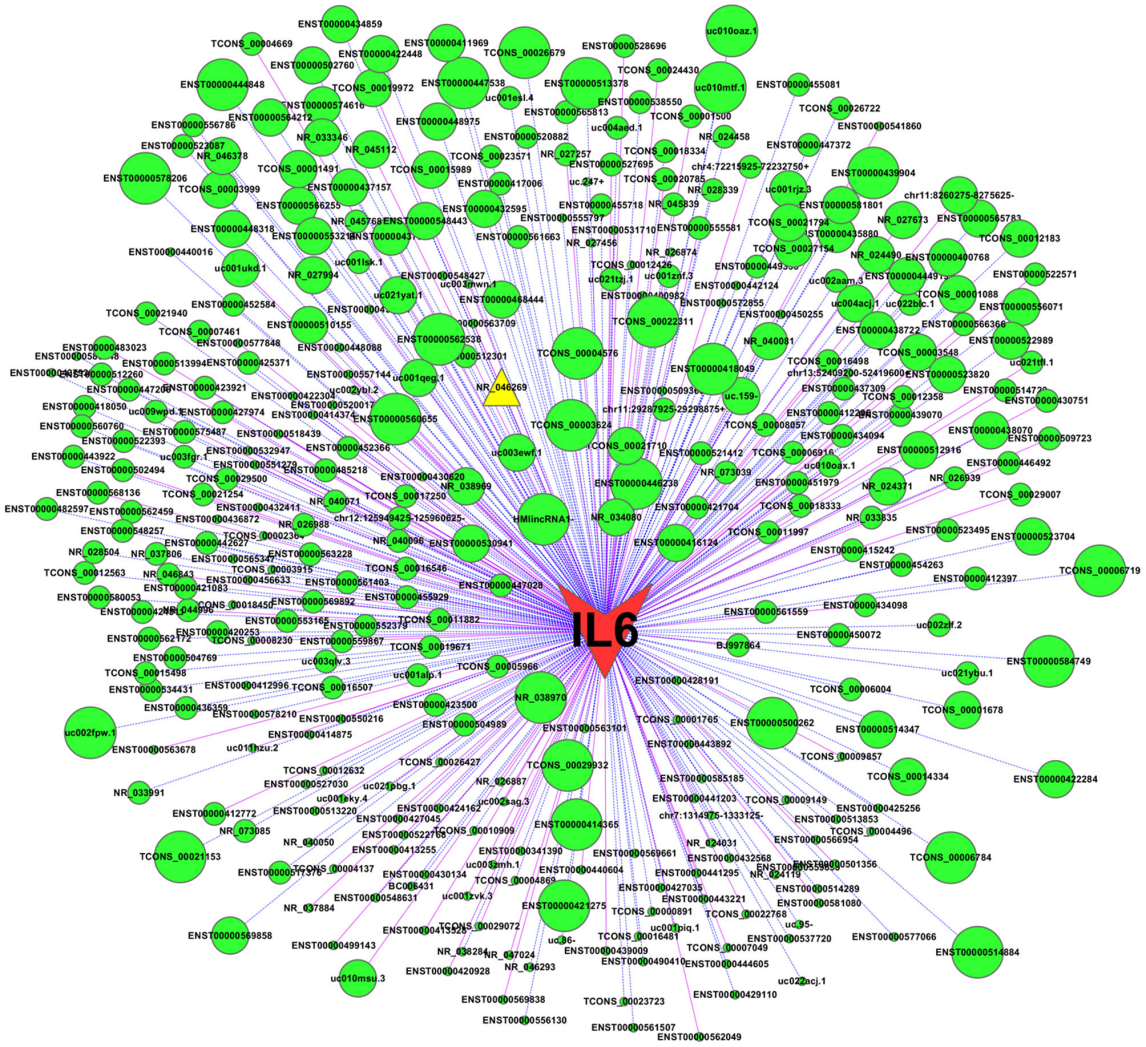
CNC network between IL-6 and differentially expressed lncRNAs. Using expression data for IL-6 mRNA and lncRNAs in TM-infected BEAs-2B cells, a CNC network was constructed using Cytoscape (v2.8.1). |PCC| ≥ 0.91 and *P* < 0.05 was identified as significant. Nodes represent correlations between IL-6 and lncRNAs, while edges represent co-expression relationships. Solid pink lines represent positive co-relationships and blue dotted lines represent negative. Larger node sizes indicate more extensive relationships between IL-6 and the lncRNAs.

To examine potential mechanisms by which IL-6 is down-regulated by TM, we constructed a CNC network between IL-6 and the differentially expressed lncRNAs. The co-expression relationship of each gene pair was estimated by the PCC. In the CNC network, IL-6 was associated with 350 lncRNAs (|PCC| ≥ 0.91, *P* < 0.05), of which 102 pairs presented as positive correlations and 248 pairs presented as negative correlations. Notably, IL-6 was highly negatively correlated with lncRNA NR_046269 (PCC = −0.998, Figure 3), indicating a potential role for this lncRNA in regulating IL-6 expression in response to TM. Since lncRNA NR_046269 is adjacent to the coding gene single stranded DNA binding protein 1 (SSBP1), and partially overlaps with its transcribed region, we designated this lncRNA as lncSSBP1.

### LncSSBP1 Negatively Regulates IL-6 Expression

**Figure 4:**
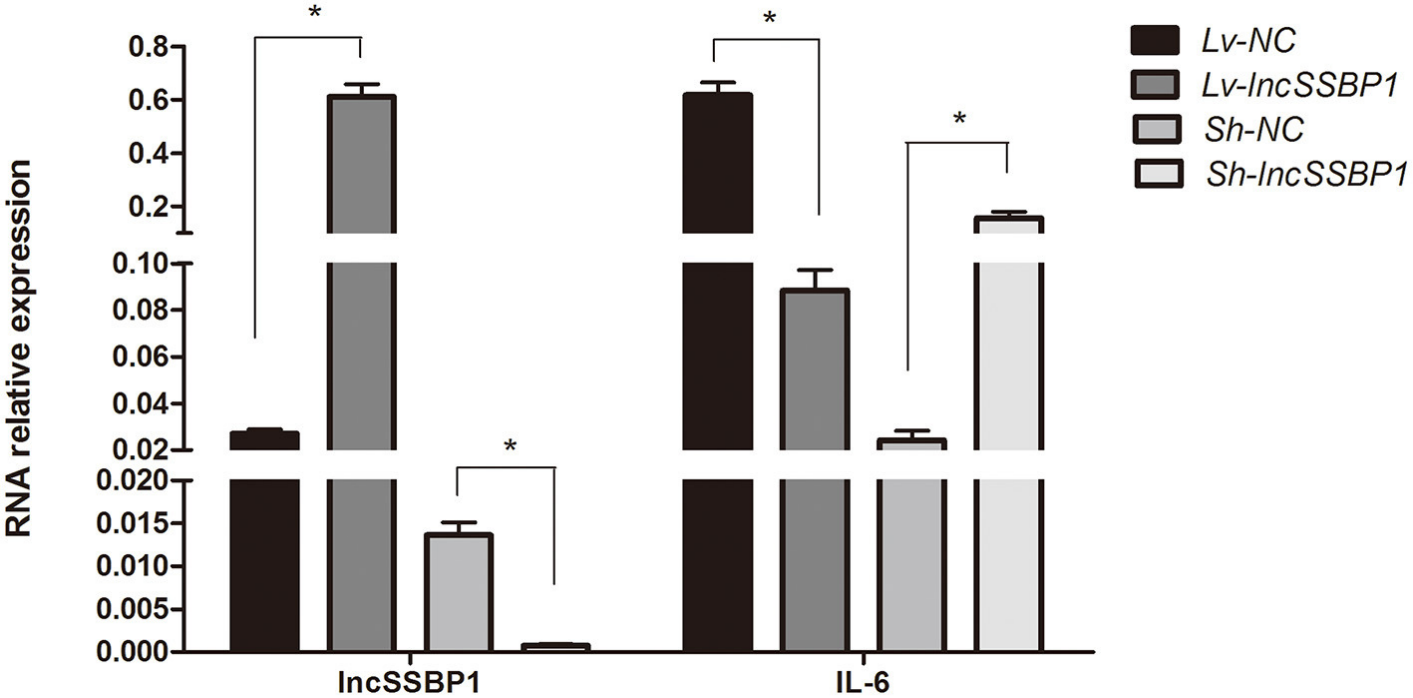
Overexpression and knock down of lncSSBP1 in BEAS-2B cells inversely regulates IL-6 expression. **(Left)** qRT-PCR was performed to verify lncSSBP1 overexpression or knockdown in response to lncSSBP1 (Lv-lncSSBP1) or control (Lv-NC) expression lentivirus, and lncSSBP1 (Sh-lncSSBP1) or control (Sh-NC) shRNA lentivirus. **(Right)** At the same time, the relative expression levels of IL-6 were detected, *P < 0.05.

To directly investigate whether lncSSBP1 regulates IL-6 expression, we used lentiviral vectors to overexpress and knock down lncSSBP1 in BEAS-2B cells. qRT-PCR results showed that the relative IL-6 mRNA expression level was significantly decreased after overexpression of lncSSBP1. Conversely, the IL-6 mRNA level was significantly increased after knock down of lncSSBP1 (*P* < 0.05, Figure 4). These results provide evidence that lncSSBP1 has a negative regulatory effect on IL-6.

### LncSSBP1 Localizes to the Nucleus in BEAS-2B Cells

**Figure 5:**
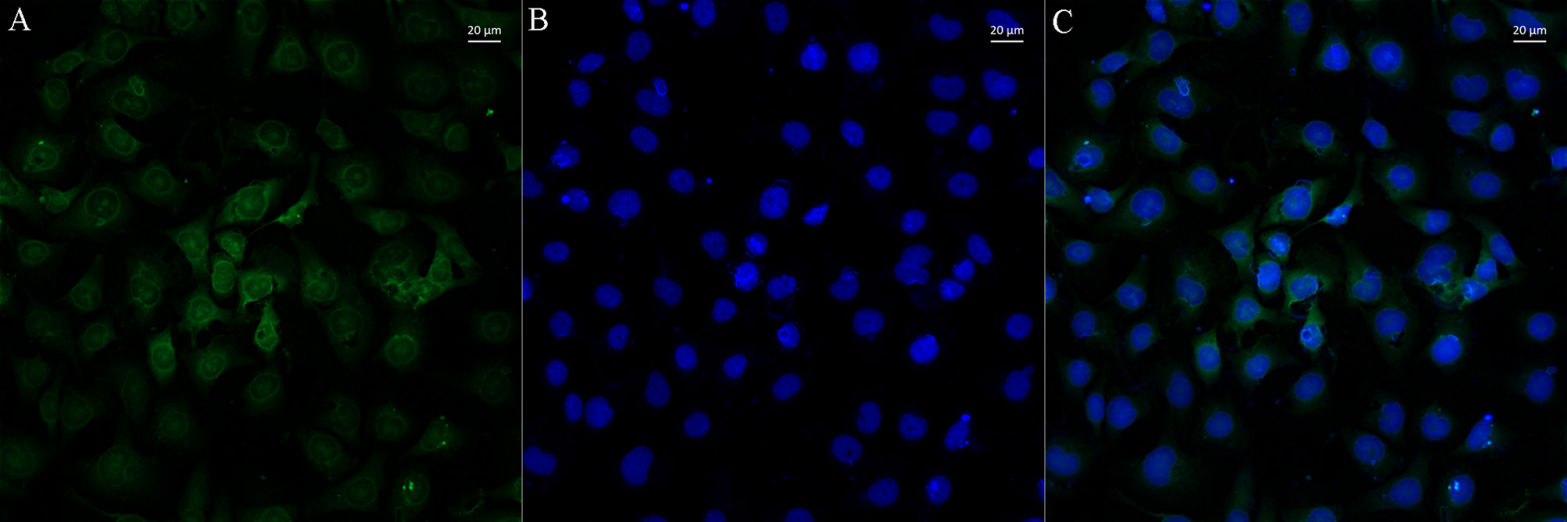
LncSSBP1 is localized to the nucleus in BEAS-2B cells. **(A)** Green fluorescence of lncSSBP1 (excitation wavelength 550 nm and emission wavelength 570 nm) was detected by laser confocal microscopy. **(B)** The nuclei were DAPI counterstained (blue color; excitation wavelength 360 nm and emission wavelength 470 nm). **(C)** Overlay of **(B)** on **(A)**.

To further explore the function of lncSSBP1, FISH was performed. The results show that lncSSBP1 was mainly distributed in the nucleus with a small amount distributed in the cytoplasm around the nucleus (Figure 5). These findings are consistent with a nuclear regulatory role for lncSSBP1.

### LncSSBP1 Is Unlikely to Regulate IL-6 by Functioning as a Competing Endogenous RNA (ceRNA)

**Figure 6:**
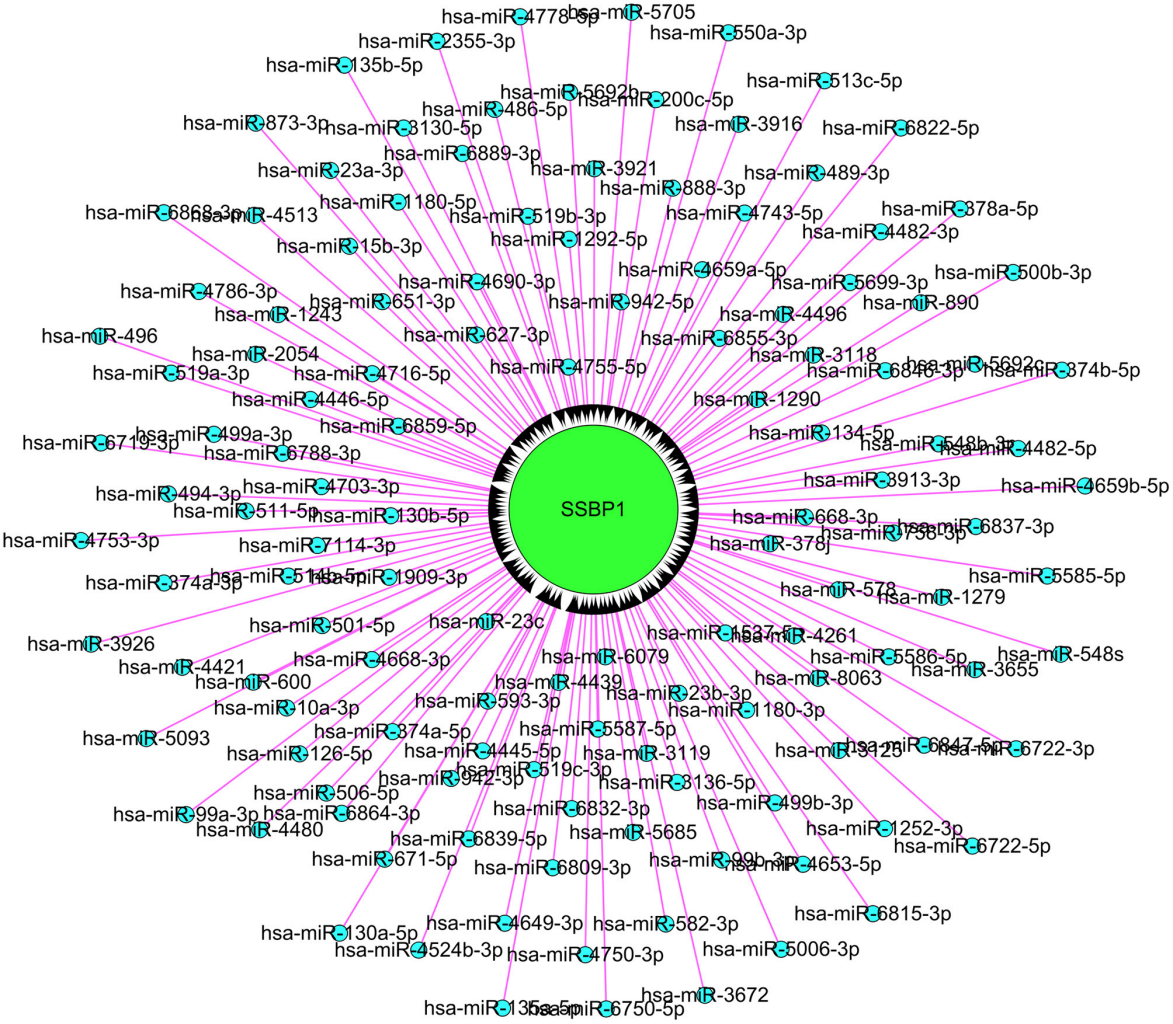
Assessment of microRNA that interact with lncSSBP1 using computational approaches. In total, 129 microRNAs were identified to interact with lncSSBP1, though none of the microRNAs targeted mRNAs that were modulated by TM infection.

Using computational approaches, we calculated the likelihood of lncRNA-microRNA-mRNA interaction. A total of 129 microRNAs were retrieved as candidate interacting partners of lncSSBP1 (Figure 6). However, none of these microRNAs were predicted to interact with differentially expressed mRNAs, including IL-6 in our profiled data. These results suggest that lncSSBP1 is unlikely to regulate IL-6 expression by functioning as a ceRNA.

### LncSSBP1 RNA Inhibits IL-6 Expression via Interaction With hnRNPK

To identify alternate methods by which lncSSBP1 may regulate IL-6 expression, we performed RNA pull down assays followed by mass spectrometry of proteins that bind directly or indirectly to lncSSBP1. In total, 284 proteins were obtained (Table S2), however, this set of lncSSBP1-interacting partners did not include IL-6 transcription factors. Therefore, we sought to identify other interactions that may affect IL-6 expression. Using computational approaches, we determined that hnRNPK is among the higher-scoring proteins. As hnRNPK belongs to a class of nuclear RNA-binding proteins that interact with precursor mRNAs (18–21), we reasoned that it could potentially mediate the function of lncSSBP1.

**Figure 7:**
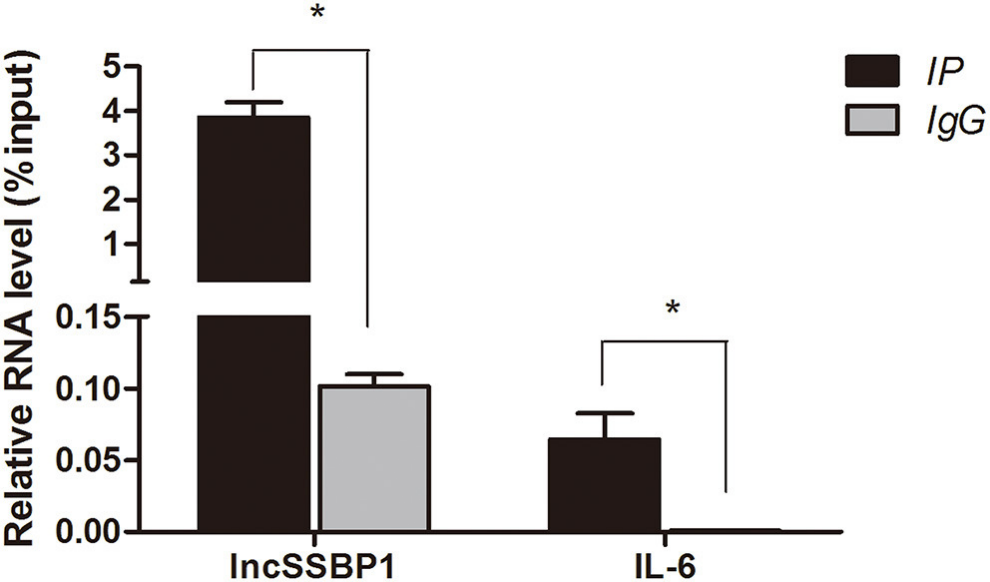
hnRNPK interacts with lncSSBP1 and IL-6 mRNA in an RNA immunoprecipitation assay. Anti-hnRNPK was used to immunoprecipitate hnRNPK complexes, which were evaluated for lncSSBP1 and IL-6 mRNA content by qRT-PCR. RNA levels are presented as relative expression compared to the input group, *P < 0.05.

To examine the specificity of the interaction between lncSSBP1 and hnRNPK in BEAS-2B cells, we performed RIP assays with anti-hnRNPK antibodies or control IgG, followed by qRT-PCR. The relative enrichment of lncSSBP1 in the IP-hnRNPK group was remarkably higher than in IgG group. Furthermore, the relative expression of IL-6 mRNA in the IP-hnRNPK group was also significantly higher than in the IgG group (Figure 7). These results indicate that hnRNPK interacts with both lncSSBP1 and IL-6, which provides a pathway by which lncSSBP1 may regulate IL-6.

## Discussion

In the process of infection, pathogens tend to induce the body to produce immunosuppressive factors to evade immune attacks, so as to promote their own survival. Immune evasion has been demonstrated in TM pathogenesis *in vivo* (22, 23), though its exact mechanisms are poorly understood. Like *M. tuberculosis*, TM is engulfed by monocytes/macrophages and lurks in the reticuloendothelial system (23). Furthermore, TM can survive oxidative stress, heat shock, nutrient starvation, pH, enzymes, and dimorphic morphologic shifts (22, 24, 25). Studies have indicated that IL-6 and its signaling pathways are involved in immune evasion (26–28). For most early stages of infections, the expression of IL-6 is elevated. However, for some infections, the expression of IL-6 is reduced. For example, *M. tuberculosis* secretes lower levels of IL-6 when infected in adipocytes, which may help *M. tuberculosis* to lurk in fat cells (29). Furthermore, for *Chlamydia trachomatis*, there are no differences in the expression levels of IL-6, TNF-α, and CXCL8 for early stage infection of cervical epithelial cells, indicating that it can also escape from the strong pro-inflammatory responses, thus facilitating its adaptation to the intracellular microenvironment (30). In our previous and present studies (15), using microarray, qRT-PCR and ELISA, we identified that IL-6 were all significantly decreased in human bronchial epithelial cells incubated with TM conidia for 4 h, while using transmission electron microscope, we observed that the number of conidia and organelles was significantly increased in the same cell model, and the Golgi, lysosomes and other organelles appeared swollen over time. So we supposed that TM induced cytoplasmic organelles injury and reduction of IL-6 may facilitate its invasion.

LncRNAs are major participants in gene expression regulatory networks, and their precise sequence and natural domains define their molecular mode of action and specific execution of their biological functions (31). They regulate transcription, splicing, nucleic acid degradation and translation through RNA-RNA, RNA-DNA, or RNA-protein interactions (32). LncRNAs have been disclosed to have critical roles in modulating immune responses, but the majority of them remain uncharacterized (33). Using microarray and CNC analysis, we found that lncSSBP1 is highly negatively correlated with IL-6 expression after TM infection, suggesting that lncSSBP1 may have a negative regulatory effect on IL-6 expression. Subsequently, cell transfection experiments demonstrated that IL-6 expression was down-regulated when lncSSBP1 was overexpressed, while IL-6 was up-regulated when lncSSBP1 was knocked down.

To explore the potential mode of action of lncSSBP1, we examined its subcellular localization. Using FISH, we determined that most of the lncSSBP1 was localized to the nucleus, with a small amount in the cytoplasm, indicating that lncSSBP1 may carry out its activities through the interaction with microRNAs (as a ceRNA), transcription factors, heterogeneous ribonucleoproteins (hnRNPs), or chromatin-modifying complexes in the nucleus.

We used computational approaches to assess the likelihood of lncRNA-microRNA-mRNA interactions. However, we did not predict any microRNA interactions with IL-6 mRNA, suggesting that lncSSBP1 is not likely to function as a ceRNA in regulating IL-6 expression.

To further evaluate potential mechanisms of lncSSBP1, we performed RNA pull down and mass spectrometry experiments of lncSSBP1 interacting proteins. We did not find IL-6 transcription factors among the proteins pulled down by lncSSBP1, and we therefore looked for other proteins that might affect IL-6 expression. Among the detected proteins, hnRNPK was identified as a candidate target that may be involved in IL-6 mRNA processing.

hnRNPK is a component of heterogeneous nuclear ribonucleoprotein complexes (hnRNPs) that interacts with precursor mRNAs and are by far the most important molecular chaperones that mediate the functions of lncRNAs (18–21). As a component of hnRNPs, hnRNPK is reported to participate in transcription, splicing, editing, localization, and degradation of mRNAs and maturation of microRNA precursors (34, 35). Additionally, there is evidence that hnRNPK contributes to the regulation of RNA 3′-end processing. In THP-1 monocytes, hnRNPK is shown to control cytoplasmic COX-2 mRNA stability by modulating its binding to the COX-2 promoter and COX-2 3′-UTR (36). RIP-Chip analyses demonstrated 1,901 mRNAs that were differentially bound to hnRNPK, which interacts specifically with a sequence in the transforming growth factor-β-activated kinase 1 (TAK1) mRNA 3′-UTR in LPS-activated macrophages. Reduction of hnRNPK increases endogenous TAK1 mRNA translation, resulting in enhancement of TNF-α, IL-1β, and IL-10 mRNA expression (37). In this report, hnRNPK was found to significantly enrich lncSSBP1 in pull down assays, indicating that lncSSBP1 binds specifically to hnRNPK. HnRNPK also bound to IL-6 mRNA, suggesting that lncSSBP1 may repress basal expression levels of IL-6 mRNA through its interaction with hnRNPK. These findings provide a pathway by which lncSSBP1 may regulate the expression of IL-6 during TM infection to enhance immune evasion.

In summary, IL-6 expression is down-regulated during TM infection in bronchial epithelial cells. LncSSBP1 has an overall negative effect on IL-6 expression, which may be beneficial to TM immune evasion. Our findings suggest that lncSSBP1 may perform its regulatory activity on IL-6 mRNA by specifically interacting with hnRNPK. Additional mechanisms by which lncSSBP1 affects IL-6 remain to be addressed in future studies.

## Data Availability Statement

All datasets generated for this study are included in the article/**Supplementary Material**.

## Author Contributions

ZH and XL designed the research, interpreted the data, and gave final approval of the version to be published. YiL contributed to computer programs, data analysis, and drafted the manuscript. YiL, HC, SL, and YuL performed the majority of the experiments and of the data collection. GL, JB, and HL provided suggestions during manuscript preparation and revised the manuscript.

### Conflict of Interest

The authors declare that the research was conducted in the absence of any commercial or financial relationships that could be construed as a potential conflict of interest.
