# Supplementary material for: LncSSBP1 Functions as a Negative Regulator of IL-6 Through Interaction With hnRNPK in Bronchial Epithelial Cells Infected With Talaromyces marneffei
Source: Front Immunol. 2020 Jan 10;10:2977. doi: 10.3389/fimmu.2019.02977 (PMC6966331; doi:10.3389/fimmu.2019.02977)
Supplement: Supplementary file 1 [file Table_1.DOCX]

**Table S1 Sequences of primers used for quantitative real time PCR**

| Gene | Primer sequence (5'-3') |
| --- | --- |
| NR_046269-Forward | ACTTGGCAGCAACTCTTAGGT |
| NR_046269-Reverse | GCCCAAGTAAGTGCACACGA |
| IL-6- Forward | AGTGAGGAACAAGCCAGAGC |
| IL-6- Reverse | ATTTGTGGTTGGGTCAGGGG |
| GAPDH- Forward | GCACCGTCAAGGCTGAGAAC |
| GAPDH- Reverse | TGGTGAAGACGCCAGTGGA |
| 7SK- Forward | GACATCTGTCACCCCATTGA |
| 7SK- Reverse | AGACCGGTCCTCCTCTATCG |
